# ETHNIC DIFFERENCES IN DIET QUALITY AND COGNITION AMONG OLDER ADULTS

**DOI:** 10.1093/geroni/igad104.1736

**Published:** 2023-12-21

**Authors:** Isabella Arellanes, Eileen Crimmins, Jennifer Ailshire, Jung Ki Kim

**Affiliations:** University of Southern California, Los Angeles, California, United States; University of Southern California, Los Angeles, California, United States; University of Southern California, Los Angeles, California, United States; University of Southern California, Los Angeles, California, United States

## Abstract

Research indicates an association between diet quality and cognitive outcomes. However, the association across ethnicities is not well understood. This study identifies differences in diet quality using the Healthy Eating Index (HEI-2015) score by ethnicity and its relationship to cognitive outcomes within ethnic groups. The sample includes 6,845 older adults from the 2014 wave of the Health and Retirement Study and nutrition data from the 2013 supplemental Health Care and Nutrition Survey. Cognitive functioning was measured based on a scale ranging from 0-27. Overall, 11% of respondents had a good quality diet, indicated by a HEI-2015 score of 81-100. Diet quality was significantly better among Hispanics than either non-Hispanic Blacks or Whites (p< 0.001 in both comparisons). Diet quality was not significantly different between Whites and Blacks (p=0.82). In a regression model controlling for age, gender, and marital status, the association between diet quality and better cognition was significant among Whites (p< 0.001) and Blacks (p< 0.05), but not significant among Hispanics (p=0.11). A one unit increase in the Healthy Eating Index is associated with a 0.05 increase in cognitive score among Whites and a 0.03 increase among Blacks. These findings suggest significant differences in diet quality across ethnicities that may impact cognition among older adults; however, the impact may differ by ethnicity.

